# Active Chemical Sampling Using Jet Discharge Inspired by Crayfish: CFD Simulations of the Flow Fields Generated by the Jet Discharge Device †

**DOI:** 10.3390/s20020522

**Published:** 2020-01-17

**Authors:** Hanako Ishida, Ryuichi Takemura, Tatsuki Mitsuishi, Haruka Matsukura, Hiroshi Ishida

**Affiliations:** 1 Graduate School of Bio-Applications and Systems Engineering, Tokyo University of Agriculture and Technology, Tokyo 184-8588, Japan; hanako.ishida05@gmail.com (H.I.); ryuichi.take@gmail.com (R.T.); t.mitsuishi0506@gmail.com (T.M.); 2 Research Fellow of Japan Society for the Promotion of Science, Tokyo 102-0083, Japan; 3 Graduate School of Engineering Science, Osaka University, Osaka 560-8531, Japan; haruka.matsu@sys.es.osaka-u.ac.jp

**Keywords:** active sensing, chemical sensor, crustacean, olfactory search behavior, biomimetics

## Abstract

Here, we report on computational fluid dynamics (CFD) simulations conducted to develop a chemical sample collection device inspired by crayfish. The sensitivity of chemical sensors can be improved when used with a sniffing device. By collecting fluid samples from the surroundings, all solute species are also collected for the sensor. Crayfish generate jet-like water currents for this purpose. Compared to simply sucking water, food smells dissolved in the surrounding water can be more efficiently collected using the inflow induced by the jet discharge because of the smaller decay of the inflow velocity with the distance. Moreover, the angular range of water sample collection can be adjusted by changing the directions of the jet discharge. In our previous work, a chemical sample collection device that mimics the jet discharge of crayfish has been proposed. Here, we report CFD simulations of the flow fields generated by the device. By carefully configuring the simulation setups, we have obtained simulation results in which the angular ranges of the chemical sample collection in real experiments is well reproduced. Although there are still some discrepancies between the simulation and experimental results, such simulations will facilitate the process of designing such devices.

## 1. Introduction

Olfaction often plays a vital role in orienting behavior, and many animals rely for their survival on this ability. For some animal species, the olfactory cues (or chemical cues in general) are far more reliable than visual or auditory cues. Male moths can follow aerial trails of sexual pheromones over long distances to find their mates [1]. Marine crustaceans such as lobsters and crabs can track the smell of food to survive in the ocean [2]. The success of the chemical orientation behavior of these animals has inspired the development of mobile robots with chemical sensing capabilities [3]. If appropriate chemical sensors are provided and effective search algorithms are implemented, robots will become able to search chemical sources autonomously like moths and marine crustaceans. The potential applications for such robots include searching for chemical leaks, hazardous items, and environmental pollutant sources.

However, there still are gaps between the capabilities of animals and state-of-the-art chemical sensing technologies. For example, trained dogs can sniff out a trace scent of explosives at ppb levels in a fraction of seconds [4]. The response time, sensitivity, and selectivity of currently available gas sensors do not match those of the dog nose. Moreover, dogs are more intelligent and agile than most robots developed so far. Various research efforts are being made to fill those gaps.

Interestingly, animals not only have keen olfaction but also have evolved highly efficient ways of collecting chemical molecules onto their olfactory organs. Not much attention has been paid to this feature of animal olfaction although efficient chemical sample collection is essential to achieve trace chemical detection [5]. For example, when mammals sniff, air is inhaled into the nostrils, and, therefore, the olfactory signal acquisition is promoted. Interestingly, dogs can deform their external nares when expiring air though their nose, so that the air is ejected ventrally and laterally backward [5]. When sniffing an object, the disturbances that the expired air may introduce to the object are kept minimized by deforming the nares and expiring backward. Moreover, the backward jet-like airflow entrains the surrounding air, and induces air currents from the front of the nose to the jet. These air currents further help dogs collect air samples to their nose [6].

**Figure 1:**
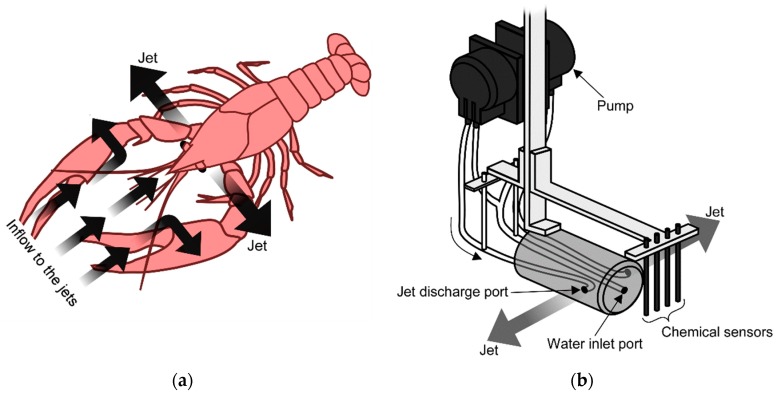
Jet-assisted water sample collection. (**a**) schematic diagram of flow induced by a crayfish as a result of lateral jet discharge. Reprinted from [21]; (**b**) schematic diagram of the device that models the jet discharge of crayfish.

Such fluid dynamic considerations are bringing new insights into our understanding of animal olfaction. The major chemoreceptor organ of aquatic crustaceans is their antennules. They have been observed to flick their antennules when they perceive chemical stimuli. This flicking is now considered to be an action to promote chemical reception on the antennules. By flicking the antennules, old water samples staying on the antennules owing to the viscosity of water are flushed out, and new water samples are brought [7]. Experiments on crayfish have revealed even more interesting behavior. A crayfish has three pairs of maxillipeds around its mouthpart below the antennules. The flagella of the exopodites of these maxillipeds have a fan-like shape. A crayfish generates jet-like water currents as shown in Figure 1a by waving the maxillipeds [8]. The flow induced by the jets is considered to help collect water samples from the surroundings to the chemoreceptors on the antennules.

This behavior is similar to the jet-assisted olfaction [5] of dogs, but has larger impact in underwater chemical sensing. The molecular diffusion in water is a hundred times slower than in air. The characteristic diffusion length in one hour is calculated to be only 5 mm [9]. Most species of crayfish live in still water environments, e.g., at the bottom of lakes and ponds, and the velocity of water flow in such environments rarely exceeds 10 mm/s [8]. Therefore, the smell of food dissolved into water mostly stays in the close vicinity of the food. Without making efforts to actively collect water samples, crayfish would perceive almost no smell when they are even slightly off from the food. Such chemical sample collection mechanism would be of great benefit if employed in underwater chemical sensing robots [10,11,12].

From an engineering point of view, the jet-assisted olfaction of crayfish has two advantages compared to simply sucking water. The first advantage is that water samples can be more quickly collected by the jet discharge [13]. When a crayfish generates jets laterally to both sides, inflow to the jets is generated as shown in Figure 1a as a result of jet entrainment. This inflow is axisymmetric around each jet, and the velocity of the inflow is inversely proportional to the distance to the jet axis. On the other hand, if water is sucked into a point sink, centrosymmetric flow field converging to the point sink is generated. In theory, the velocity of this flow is proportional to the inverse square of the distance to the point sink. Therefore, the flow generated by a point sink decays more rapidly. The second advantage is that the angular range of water sample collection can be adjusted by changing the direction of jet discharge [13]. In theory, the flow field generated by a point sink is centrosymmetric. Therefore, when water is sucked into a small inlet port, the generated inflow shows almost no change even if the orientation of the inlet port is changed.

In order to investigate the jet-assisted olfaction in crayfish, we have developed a jet discharge device as shown in Figure 1b [14]. Crayfish are known to generate jets in various directions including perpendicularly to both sides, 45° backward, and 45° upward. Four electrochemical sensors were aligned in front of the device, and their response was measured to evaluate the angular range of water sample collection for these jet discharge patterns. We also conducted computational fluid dynamics (CFD) simulations to obtain insights useful for developing underwater chemical sensing robots. However, it was found that the results of the simulations do not coincide well with the experimental results. Moreover, it was laborious to evaluate the angular range of chemical sample collection by moving a chemical source to various locations and checking the sensor response for each source location. Chemical solution drawn to the device forms a thin filamentous streak, which often passed through a gap between the sensors without touching their surface. In such cases, the chemical sensors show no response even though the chemical was drawn to the device.

In this paper, we report CFD simulations with revised simulation setups on the flow fields generated by a jet discharge device. The simulation results were compared with the results of flow visualization, instead of just measuring the response of chemical sensors placed at a limited number of positions. By carefully configuring the simulation setups, we have obtained simulation results in which the angular range of the chemical sample collection in real experiments is well reproduced. The simulation techniques described in this paper can be used to facilitate the process of designing underwater chemical sensing systems. The trajectory of chemical solution released from a source can be predicted using the flow field data obtained in the simulations. This will enable, for example, the investigation of the optimum sensor arrangement in front of a jet discharge device.

Turbulent jets have found many industrial applications, and extensive studies have been conducted on their properties [15]. However, with regard to mass and heat transport, attention has been paid mostly to cooling and cleaning. Blowing chemicals or heat off from objects using impinging jets has been extensively studied [16]. On the contrary, only a handful of investigations have been done to use jets for collecting chemical samples on sensors. Studies have shown that the reach of a suction inlet can be increased by discharging auxiliary jets to the sides from the inlet port [17]. Settles proposed to use such a device as a sniffer for chemical sensors [5]. In [18], an auxiliary jet flow is used to generate swirling flow. This cyclonic flow acts like an extended suction tube. A synthetic jet was also applied to deliver chemical samples to a gas sensor [19]. The most distinctive feature of our model animal, i.e., crayfish, is that they change directions of multiple jets to change the direction of the inflow. To the best of our knowledge, this point has been raised only in a limited number of papers including our previous conference paper [8,13,14].

The rest of the paper is organized as follows. In Section 2, details of the CFD simulation methods are described after briefly explaining the structure of the jet discharge device and the setup of the flow visualization experiments. In Section 3, the results of the revised CFD simulations are compared with previous ones, and are validated against empirical velocity profile equations for a round jet. Then, the simulation results are compared with the results of the experiments with regard to the angular range of water sample collection. This paper is based on two conference papers: the initial results of the flow visualization experiments and simulations were first presented in [20] and [21], respectively. The comparison of the simulation results with the empirical equations and flow visualization results are newly added in this paper.

## 2. Materials and Methods

### 2.1. Jet Discharge Device and Experimental Setup

The jet discharge device reported in our previous work [14] was used to model the jet generation by a crayfish. Its schematic diagram is shown in Figure 1b. The body of the device is made of a Plexiglas cylinder with a diameter of 30 mm. As shown in the figure, the device is equipped with two pumps. Water is sucked by these pumps from an inlet port with a diameter of 2.84 mm on the front side of the device. The pumped water is then discharged laterally from a pair of outlet ports with a diameter of 2.84 mm. The rate of water discharge from each outlet port is adjusted to 150 mL/min [14] so that the velocity of the inflow was matched with that of a real crayfish [13]. Three pairs of outlets ports with different nozzle angles, i.e., perpendicularly to both sides, 45° backward, and 45° upward, are aligned on the sides of the jet discharge device. The jet discharge device is also equipped with electrochemical sensors to detect the chemical substance drawn from the surroundings. As shown in Figure 1b, a laterally aligned array of four carbon rods (0.9 mm in diameter and 60 mm in length) is placed 10 mm off from the front side of the device, which serves as working electrodes of the electrochemical sensors. A piece of silver wire and a stainless-steel tube are used as a reference electrode and a counter electrode, respectively.

In the experiments, a water container (800 mm wide × 1200 mm long) was filled with water to a depth of 100 mm. The jet discharge device was placed in the middle of a water container such that the inlet port of the device came to the middle of the water container at a height of 25 mm. The height is defined here as the distance between the center of cylindrical body of the device and the bottom of the water. In our previous work [14], water sample collection by the jets was investigated by using the chemical sensors. A chemical source was placed at various locations around the jet discharge device, and the response of the four chemical sensors were observed to see if the released chemical was drawn to the device and detected by either one of the sensors. However, the chemical solution released from the source formed a thin streak when drawn to the jet discharge device. This chemical streak often passed through the gaps between the sensors without touching any of the sensors. Because the sensors showed no response in such cases, it was often unable to determine whether or not the chemical solution was drawn to the jet discharge device. In the experiments newly conducted for this paper, a line of 17 tubes was placed in front of the jet discharge device with a spacing of 10 mm to investigate the effects of jet discharge more comprehensively. Aqueous solution of fluorescent ink (Rhodamine 6G) was released from these tubes at 30 s after turning on the pumps for jet discharge. By introducing dye streaks in the flow, the flow field around the jet discharge device was visualized. The angular range of water sample collection from the surroundings to the electrochemical sensors was measured by visually observing the development of the streaklines of the ink.

### 2.2. Details of Simulations

In this paper, the results of the CFD simulations are compared with those reported in our previous work. Here, we first describe the setups of the revised simulations. A commercially available CFD software package, ANSYS Fluent 14.5 (Canonsburg, PA, USA), was used to calculate the flow fields generated by the jet discharge device. Simulations were conducted for two scenarios: jet discharge perpendicularly to both sides and 45° backward. In both cases, it was assumed that the jet discharge device was immersed in open water with a depth of 100 mm. As in the experiments, the jet discharge device was placed such that its inlet port came to the middle of the calculation domain at a height of 25 mm. Water was sucked into the frontal inlet port, and was ejected at 150 mL/min from each jet discharge port with a turbulent intensity of 10%. The diameter of all inlet and discharge ports was 2.84 mm. The no-slip boundary conditions were applied to the bottom of the calculation domain and the walls of the jet discharge device. The free-slip conditions were applied to the top surface of the calculation domain to simulate water surface. The open boundary conditions with a constant pressure were applied to the lateral sides of the calculation domain. By letting water freely move through those open boundaries, the flow fields generated in open water with no sidewall were simulated.

**Figure 2:**
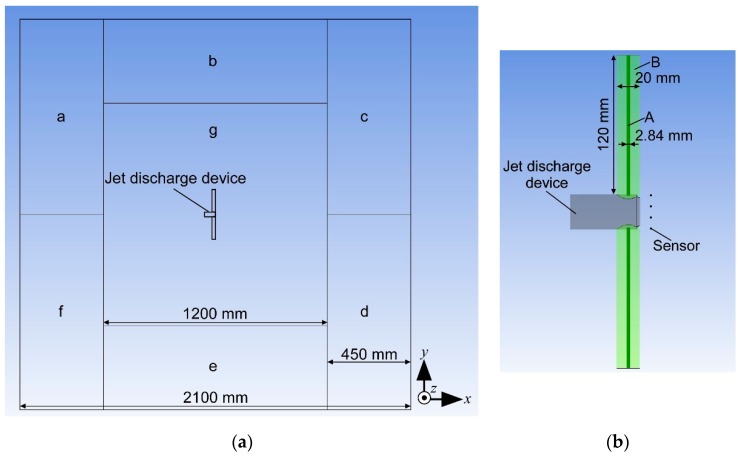
(**a**) division of calculation domain for setting grids with different mesh sizes. Structured grids with relatively coarse mesh were generated in regions a–f. In region g, a fine unstructured grid was generated around the jet discharge device; (**b**) enlarged view around the jet discharge device. Cylindrical region A has a diameter of 2.84 mm and extends from each jet discharge port. The diameter of region B that wraps around the jet is 20 mm. The four dots in the figure indicate the locations to place chemical sensors.

**Figure 3:**
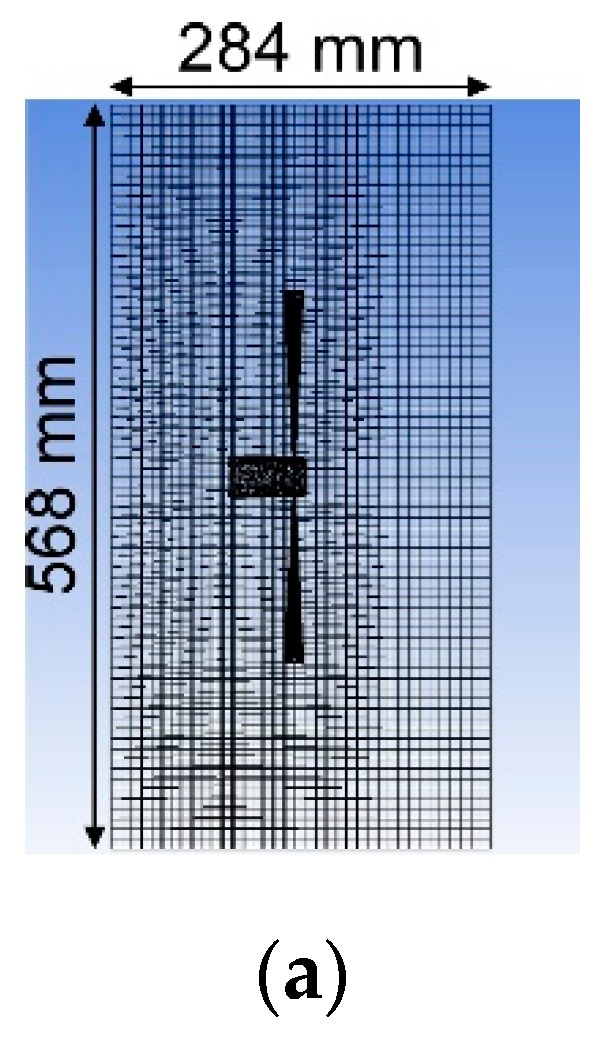

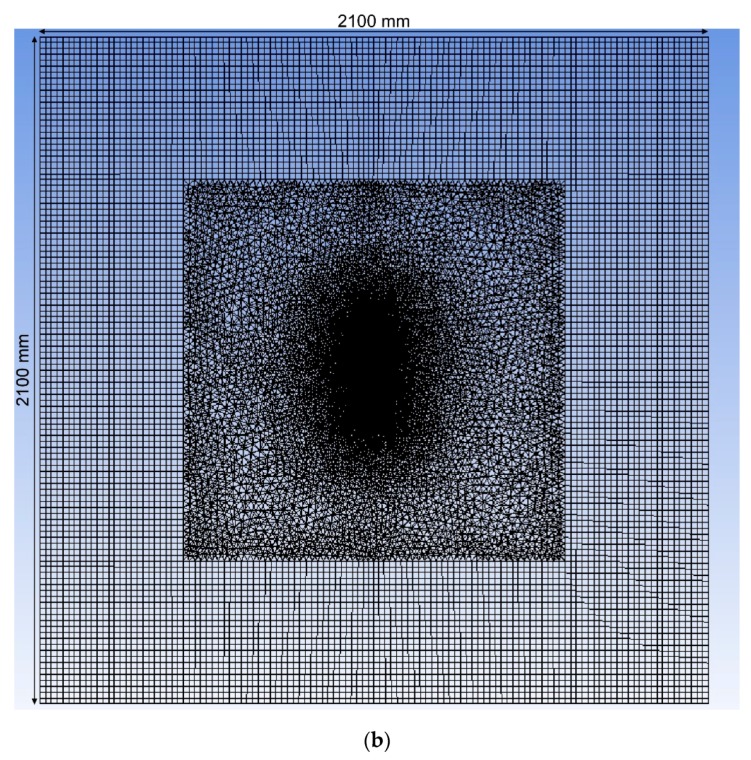
Comparison of the meshes for simulating jet discharge perpendicularly to both sides. (**a**) mesh used in the previous simulation. Note that this is not the expanded view around the jet discharge device: the whole calculation domain (only 284 mm × 568 mm) is shown in the figure; (**b**) mesh generated for the revised simulation. The size of the calculation domain was increased to prevent the calculation results from being affected by the domain boundaries.

Figure 2 shows the division of the calculation domain for generating grids with different mesh sizes in the newly conducted simulations. Figure 3b shows the generated mesh for simulating perpendicular jet discharge to both sides with the revised setups. Structured grids with a relatively coarse mesh size (approximately 20 mm) were generated in regions a–f in Figure 2 along the lateral boundaries of the computational domain. A fine unstructured grid was generated in region g to have fine mesh around the jet discharge device. The mesh size was gradually inflated at a rate of 1.06 to have smooth transition to the surrounding regions. Fine mesh was also generated along the jets so that high velocity gradients between the jets and surrounding water could be fully reproduced in the simulations. A pair of coaxial cylindrical regions A and B was defined in the computational domain for each jet, as shown in Figure 2b. Region A extends from each jet discharge port in the direction of the port opening. The diameter of region A (2.84 mm) was matched with the diameter of the jet discharge port. The finest grid with a mesh size of 0.284 mm was generated in this jet core region. The outer diameter of region B was set to 20 mm. The mesh size was gradually changed in this region: the size of the innermost cells in region B was matched to the cell size in region A, and the size of the outermost cells was matched to the cell size in region g. The length of regions A and B were set to 120 mm for jets discharged perpendicular to the side and 125 mm for jets discharged 45° backward.

Major changes in the current simulation setups from the previous ones were listed in Table 1 and Table 2. In our previous work [14], large eddy simulations (LES) were conducted using a dynamic Smagorinsky model, in which the Smagorinsky constant is dynamically calculated [22]. Figure 3a shows the mesh used in the previous simulation for perpendicular jet discharge to both sides. The size of the computational domain for jet discharge to the sides was only 568 mm in width, 284 mm in depth, and 100 mm in height. The size of the domain for jet discharge to 45° backward was 284 mm in width, 284 mm in depth, and 100 mm in height. As shown in Figure 3a, a conical region with a finer grid was placed along each jet. Its length was 127 mm, and its diameter was varied from 2.84 mm on the proximal side to 14.2 mm on the distal side.

When we conducted the flow visualization experiments, however, we found significant discrepancies between the previously conducted CFD simulations and experimental results. This led us to revise the simulation setups. The simulation of jet discharge in stagnant water is much more challenging than it appears to be. A fine computational grid is required to fully reproduce steep velocity gradients between a jet and surrounding water. Moreover, the flow in a jet is highly unstable. Even if the flow discharged from a nozzle is laminar, it soon becomes turbulent. Most turbulent models used in CFD simulations have been developed for fully developed turbulent flow, and, therefore, produce errors when used for calculating the jet flow in laminar-turbulent transition regions [23].

After many trials and errors, the size of the computational domain and the number of cells have been increased as shown in Figure 3. However, the computational cost has also increased significantly. Therefore, the steady-state Reynolds averaged Navier–Stokes (RANS) technique [22] was adopted in the revised simulations to reduce the computational cost. In the previous LES simulations, transient features in the development of the jets after the start of the discharge were calculated. In the revised simulations, mean properties of the flow fields in the final steady state were calculated to reduce the computational cost. The realizable *k*-*ε* turbulence model was used in these RANS simulations because this model is known to generate better simulation results for simulations of a wide range of flows including round jets [24]. The default models constants [24] were used here: *C*_2_ = 1.9; *σ_k_* = 1.0; *σ_ε_* = 1.2.

### 2.3. Velocity Plofiles in Round Jet

In order to check the validity of the simulations, the velocity profiles in the simulated jets were compared with those reported in the literature [25,26,27]. Assume that water is discharged from a circular opening of a nozzle with a constant velocity over the whole cross section of the opening. Since the turbulent mixing occurs at the boundary of the jet, the velocity starts to decay from the perimeter of the jet. However, over some distance (typically 10–20 times the diameter of the opening), the mean centerline velocity of the jet is kept the same as the jet discharge velocity. This region of the jet is called the zone of flow establishment (ZFE). Once the turbulent mixing has reached the jet axis, the mean centerline velocity starts to decay. The region beyond this point is named as the zone of established flow (ZEF).

In the ZEF of a round jet, Gaussian-like velocity profiles are established for the mean axial velocity distribution [25,26,27]. Thus, the axial mean velocity $\bar{u}$ at axial distance *x* from the nozzle and radial distance *r* from the jet centerline can be expressed as

(1) $$\frac{\bar{u}}{{\bar{u}}_{m}}=\exp\left(-C_{1}\eta^{2}\right),$$

where ${\bar{u}}_{m}$ is the mean maximum velocity, η=r/x, and *C*_1_ is the empirically determined constant. The decay of ${\bar{u}}_{m}$ in the ZEF is shown to be

(2) $$\frac{{\bar{u}}_{m}}{U}=C_{2}{\left(\frac{x-x_{0u}}{d}\right)}^{-1},$$

where *U* is the jet discharge velocity and *d* is the diameter of the nozzle [25,26,27]. *C*_2_ is again the empirically determined constant, and *x*_0*u*_ is a virtual origin of the velocity. The values of these two constants are dependent on the conditions of the nozzle exit. As ${\bar{u}}_{m}$ decays, the width of the jet grows almost linearly with the distance [25,26,27]. If we define the mean half radius $r_{0.5}$ as the radial distance at which the mean axial velocity becomes ${\bar{u}}_{m}/2$, its growth can be expressed as

(3) $$\frac{r_{0.5}}{d}=C_{3}\frac{x-x_{0r}}{d},$$

where *C*_3_ is another empirically determined constant and *x*_0*r*_ is a geometrical virtual origin.

In Section 3.1, we compare the velocity profiles of jets in a simulation results to those calculated from Equations (1)–(3). For a round jet generated by discharging water at 150 mL/min from a nozzle with a diameter of 2.84 mm, Reynolds number *Re* = *U d*/*ν* is calculated to be 1117, where *ν* is the kinematic viscosity of water. Although the behavior of a jet slightly changes with the Reynolds number, the values of constants in Equations (1)–(3) are almost similar for jets with a wide range of Reynolds number. Therefore, the values obtained for a jet with a Reynolds number of 86,000 [25] were used to check the validity of the simulation results in Section 3.1 (*C*_1_ = 76.5, *C*_2_ = 5.6, *x*_0*u*_ = 3.7*d*, *C*_3_ = 0.095, and *x*_0*r*_ = 0).

## 3. Results

### 3.1. Simulation Results

**Figure 4:**
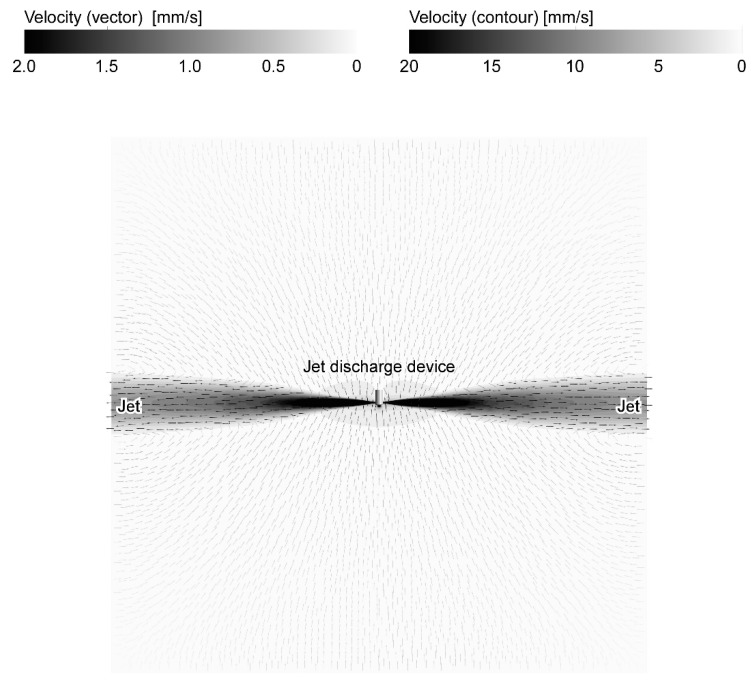
Results of the revised simulation for the jets discharged perpendicularly to both sides. The flow field on the horizontal cross section of the whole calculation domain is shown.

**Figure 5:**
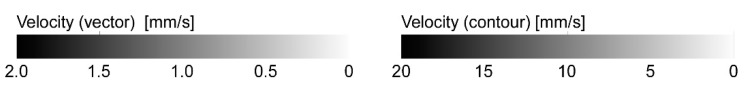

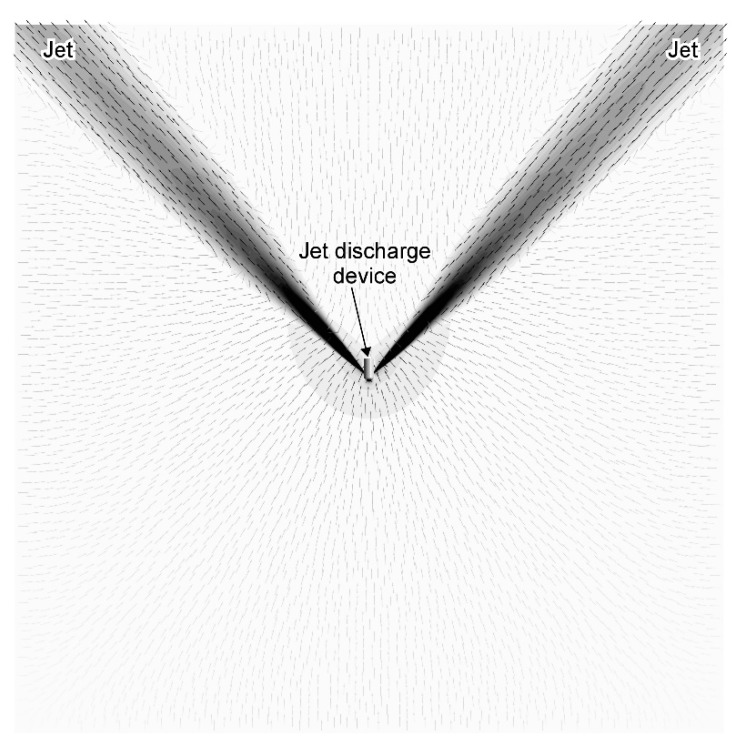
Results of the revised simulation for the jets discharged 45° backward to both sides. The flow field on the horizontal cross section of the whole calculation domain is shown.

**Figure 6:**
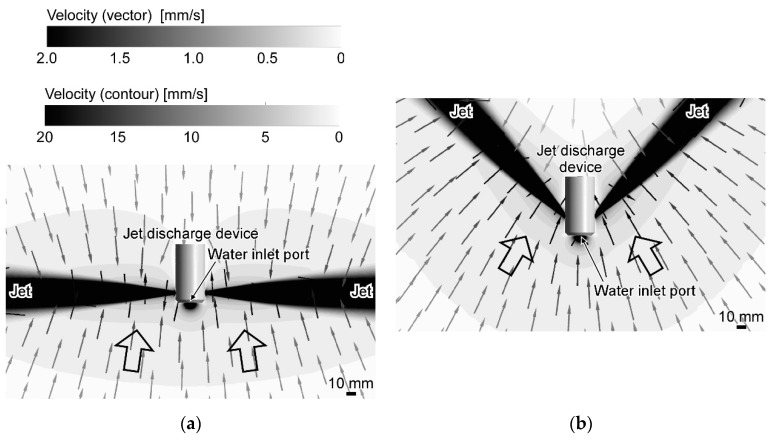
Enlarged views of the results of the revised simulation. (**a**) jets discharged perpendicularly to the sides; (**b**) jets discharged 45° backward. Large arrows show general trends of the flow; Reprinted from [21].

Figure 4 and Figure 5 show the flow fields calculated in the revised CFD simulations for two characteristic jet discharge directions, i.e., perpendicularly to both sides and 45° backward. The calculation time was 240 h and 295 h, respectively, using 2.26 GHz Intel Xeon Processor E5520. The cylinder in those figures represents the jet discharge device. The flow directions on a horizontal plane passing through the center of the jet discharge ports are shown using the small arrows. The value of the flow velocity is shown using the color of the arrows and contour. Figure 6 shows the enlarged view around the jet discharge device. For both jet discharge directions, inflow to the jet discharge device was generated. If a chemical source is placed in front of the jet discharge device, the water currents induced by the jet discharge will bring the chemical to the jet discharge device.

When a single jet is discharged, surrounding water is entrained into the jet. As a result, water currents that almost perpendicularly point to the jet axis are generated. When two jets are discharged, the flow induced by each jet is superimposed with each other. On the other hand, water suction from the frontal inlet port generates a centrosymmetric flow field converging to the suction port. However, since the velocity decay of this flow is larger, the suction has small impacts on the entire flow filed except in the vicinity of the inlet port. Thus, the flow fields shown in Figure 4, Figure 5 and Figure 6 are generated. In most places in Figure 6, the direction of the flow points almost perpendicularly to a nearby jet (see large arrows in Figure 6). The effect of suction can be seen only in the increased velocity (i.e., the darker color in the contour plots) in the small region around the inlet port.

**Figure 7:**
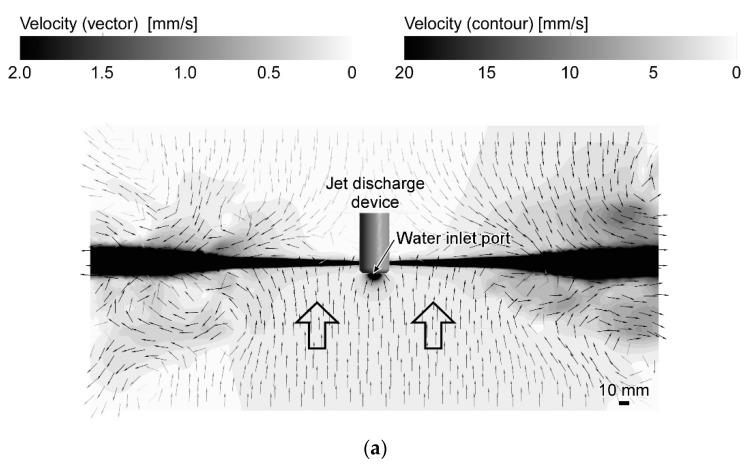

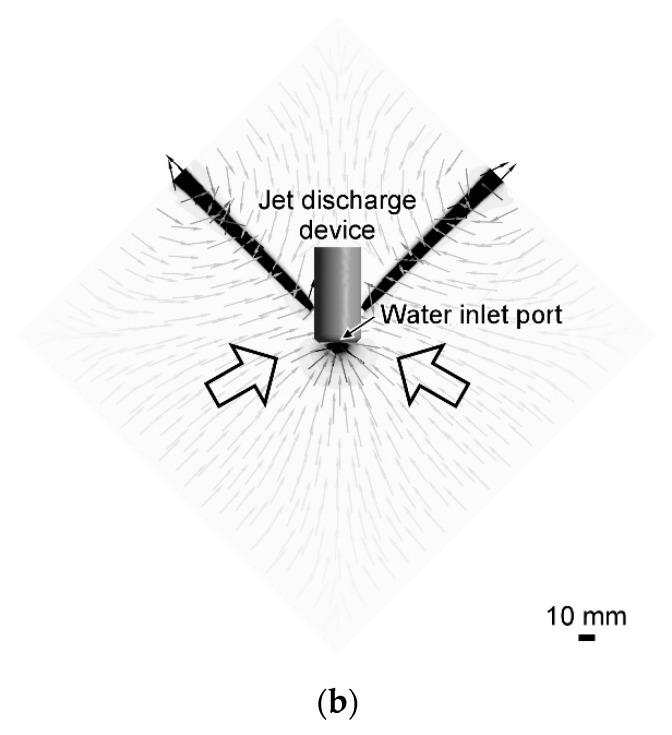
Results of the previous simulations. (**a**) jets discharged perpendicularly to the sides; (**b**) jets discharged 45° backward to the sides. Large arrows show general trends of the flow.

Figure 7 shows the results of a CFD simulations with the previous setups for jet discharge perpendicularly to the sides and 45° backward. The calculation time was 33 h and 9 h, respectively. It can be seen in Figure 7a that there are some large swirls around the distal part of the jets, making the flow field asymmetric. It should be noted, however, that a snapshot of the instantaneous flow field was calculated in this transient LES simulation. In RANS simulations shown in Figure 4, Figure 5 and Figure 6, mean properties of the steady-state flow were calculated. In addition to the swirls, however, there are also several differences between the flows in Figure 6 and Figure 7. First of all, the widths of the jets shown in Figure 7 are much smaller than those in Figure 6. In Figure 7a, the flow in the frontal region (the bottom part of Figure 7a) is apparently faster than on the backside of the device. On the other hand, flow with almost similar velocities is generated on both front and rear sides of the device in Figure 6a. In Figure 7b, the effect of the limited size of the calculation domain can be seen. Streamlines near the upper left and right boundaries in Figure 7b are unnaturally curved in such a way that the flow comes into the calculation domain always perpendicularly through these boundaries. Moreover, water suction appears to have stronger impacts in Figure 7b. As large arrows in Figure 7b shows, water is being sucked into the frontal inlet port from a wider angular range than in Figure 6b.

**Figure 8:**
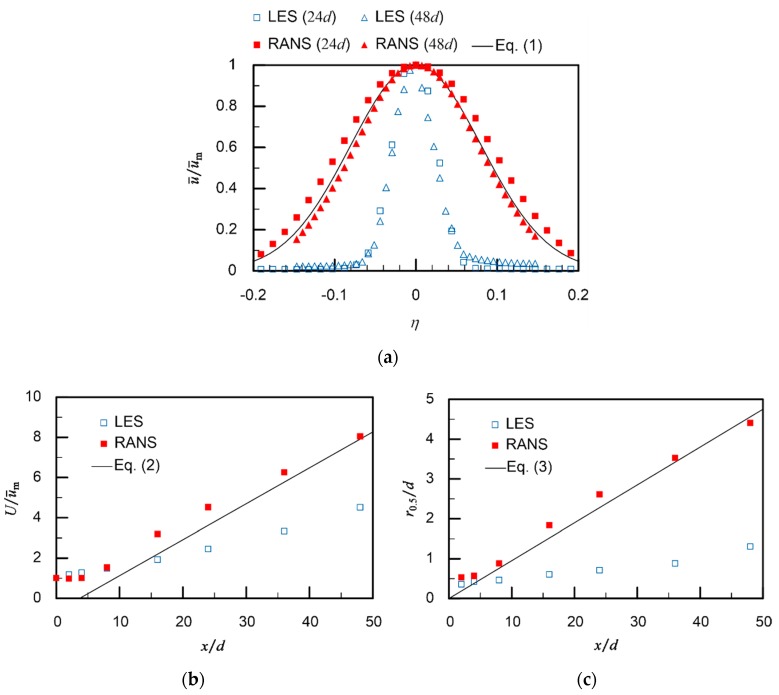
Comparison between simulation results and empirical formula. (**a**) radial profiles of the axial velocity at two different distance from the outlet port (*x* = 24*d* and 48*d*); (**b**) linear relationship between the inverse of the mean maximum velocity and the distance from the outlet port; (**c**) growth of the mean half width with the distance from the outlet port.

In order to compare the validities of the previous and revised CFD simulations, velocity profiles of the calculated jets were compared with Equations (1)–(3). Figure 8a shows the radial cross-sectional profiles of the mean axial velocity. The velocity profiles at two different distances from the jet discharge port (*x* = 24*d* and 48*d*) are plotted for the perpendicularly discharged jets. In the ZEF of a round jet, the cross-sectional velocity profile is self-similar: the normalized velocity profiles at different distances are well described by the same Gaussian distribution equation. As shown in Figure 8a, the results of the revised RANS simulation show significantly better agreement with Equation (1) than the previous LES simulation. It can be seen from Equation (2) that there is a linear relationship between the inverse of the normalized mean maximum velocity, *U*/${\bar{u}}_{m}$, and the normalized distance, *x*/*d*. As shown in Figure 8b, the result of the revised RANS simulation shows better agreement with the linear relationship derived from Equation (2). Figure 8c shows the growth of the mean half radius. Again, the result of the revised RANS simulation shows better agreement with Equation (3).

**Figure 9:**
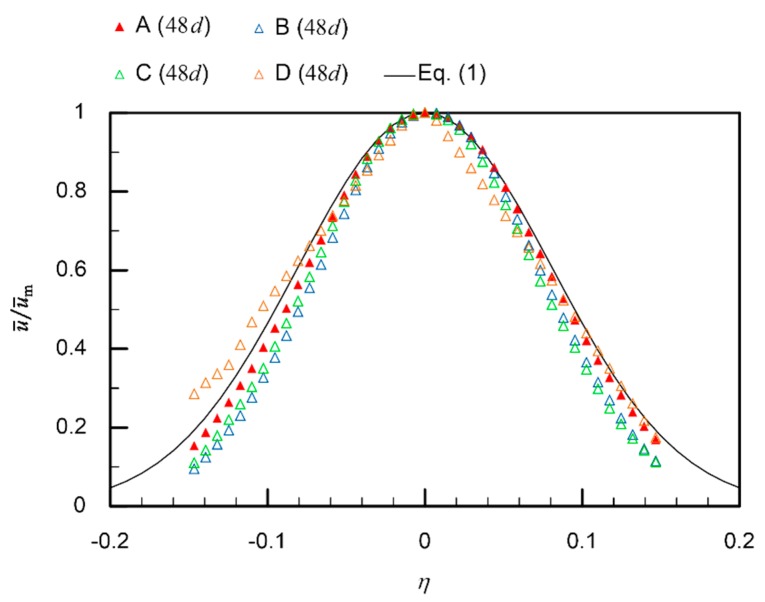
Radial velocity profiles at a distance of 48*d* from the jet discharge port. The results of RANS simulations obtained with different numbers of cells are compared. A: 11,247,418 cells (current setup); B: 4,993,844 cells; C: 2,466,709 cells; D: 524,200 cells.

These results suggest that a flow field generated by placing a jet discharge device in a shallow wide-open water can be well reproduced by a steady-state RANS simulation. In the revised simulation setups, the calculation domain was enlarged to 739 times the diameter of the jet discharge port, *d* = 2.84 mm, while the finer mesh with the size of *d*/10 was generated in the jet core regions. In Figure 9, the radial velocity profiles obtained with different mesh sizes are compared. Meshes with larger grid sizes (and therefore with smaller numbers of cells) were prepared by changing the cell growth rate from 1.06 to 1.15. The root mean square errors between the simulation results and Equation (1) were 2.8%, 7.7%, 7.2%, and 4.9% for the cases of 11,247,418, 4,993,844, 2,466,709, and 524,200 cells, respectively. The best match was obtained with the mesh used in the present simulation (11,247,418 cells). The coarser meshes produced either a skewed velocity profile (524,200 cells) or narrower profiles (4,993,844 and 2,466,709 cells).

It should be noted that, in theory, a transient LES simulation produces a thinner jet with a higher maximum velocity than the mean velocity profiles described by Equations (1)–(3). Since the velocity and shape of a turbulent jet show fluctuations, a time-averaged jet produced in RANS simulations is wider and has a lower maximum velocity. However, not much fluctuation is seen in the jet shapes in Figure 7. Therefore, large discrepancies will remain between Equations (1)–(3) and the results of the LES simulations even though the time-averaged velocity profiles are calculated from the LES simulation results.

### 3.2. Comparison with Experimental Results

**Figure 10:**
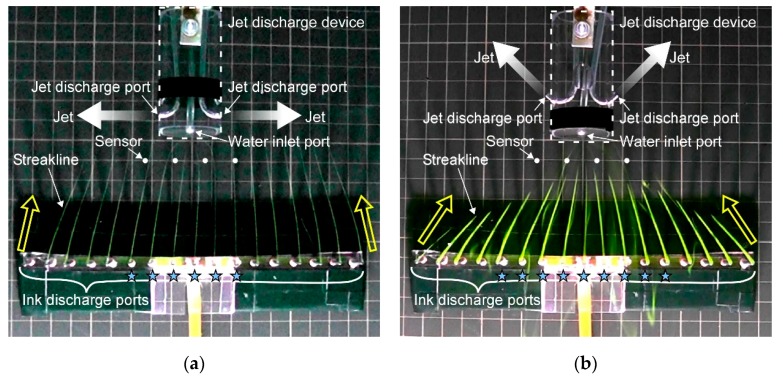
Streaks of fluorescent ink released from a line of ink discharge ports when jets were discharged (**a**) perpendicularly to the sides and (**b**) 45° backward. Positions to place electrochemical sensors are marked as white circles. Stars indicate the ink discharge ports from which ink streaks were drawn to the jet discharge device and passed between the leftmost and rightmost sensors. A 10 mm grid rubber sheet was laid on the bottom of the water container. © 2017 IEEE; Reprinted, with permission, from [20].

In this section, the results of the revised RANS simulations are further validated with the results of the flow visualization experiments. Figure 10a shows the flow field generated by the jet discharge perpendicularly to the sides. The streaks of the fluorescent ink were drawn straight to the jets. Therefore, only the water samples in the limited frontal region were drawn to the jet discharge device. Ink streaks released from six ink discharge ports that are marked by stars reached the line segment where the four chemical sensors are aligned. If a chemical source is placed in this angular range, the released chemical will be detected by either one of the chemical sensors. Figure 10b shows the generated flow field when the directions of the jet discharge were changed to 45° backward. In this flow field, the streaks of the fluorescent ink released from a wider region were drawn and converged to the jet discharge device. In this case, nine streaklines passed the line segment between the leftmost and rightmost chemical sensors.

**Figure 11:**
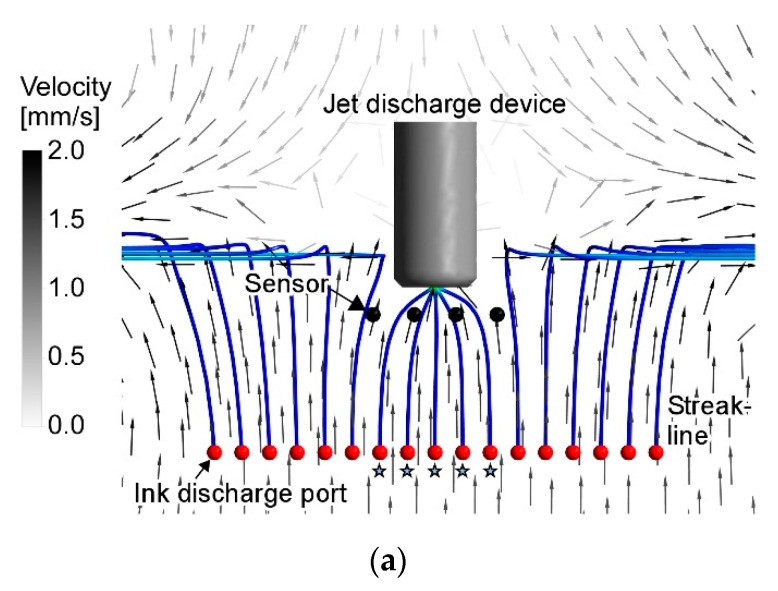

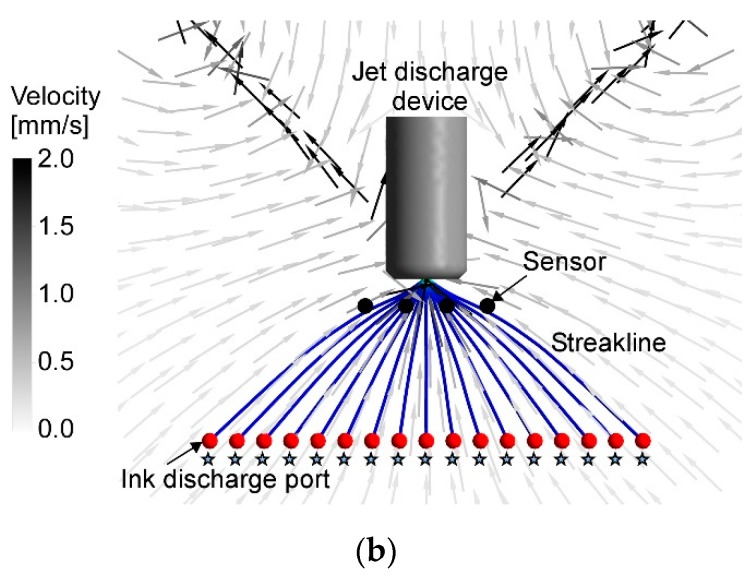
Streaklines calculated from the results of the previous simulations when jets were discharged (**a**) perpendicularly to the sides and (**b**) 45° backward. The ink discharge ports are aligned in the same way as in the experiments, and the streaklines originating from these ports are depicted. The positions of the chemical sensors are also the same as in the experiments. Stars indicate the ink discharge ports from which ink streaks were drawn to the jet discharge device and passed between the leftmost and rightmost sensors.

**Figure 12:**
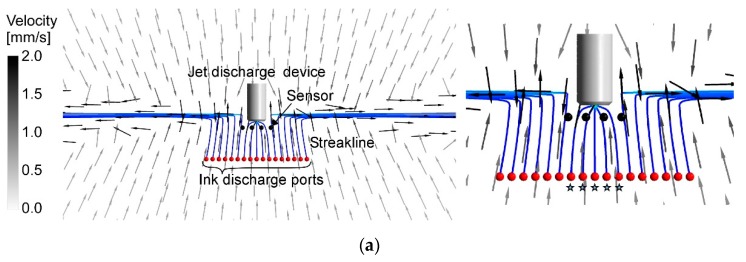

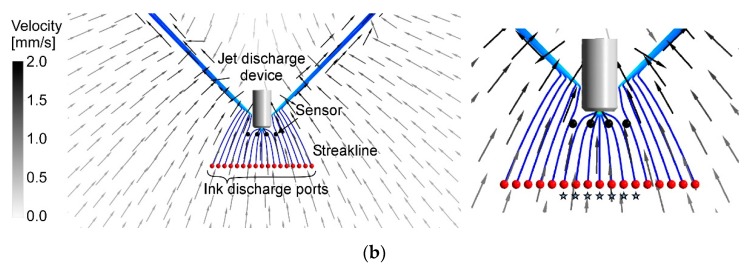
Streaklines calculated from the results of the revised simulations when jets were discharged (**a**) perpendicularly to the sides and (**b**) 45° backward. The sensors and ink discharge ports are aligned in the same way as in the experiments. Insets on the right show the enlarged views around the jet discharge device. Stars indicate the ink discharge ports from which ink streaks were drawn to the jet discharge device and passed between the leftmost and rightmost sensors.

In Figure 11 and Figure 12, streaklines calculated from the simulation results are shown, assuming that the ink discharge ports are aligned in front of the jet discharge port as in the experiments. Figure 11a shows the streaklines produced in the previous simulation result for the jet discharge perpendicularly to the sides. Five streaklines reached the jet discharge device through the array of four sensors, which agrees well with the experimental results shown in Figure 10a. However, the previous simulation for the jet discharge 45° backward produced results that were completely different from the experiment. As shown in Figure 11b, all 17 streaklines passed through the array of sensors and converged to the inlet port. The entrainment of the surrounding water into the jets appears to be underestimated in this simulation. The flow field generated in front of the jet discharge device in this simulation is closer to that generated by water suction alone.

The revised simulations produced flow fields much closer to those observed in the experiments. In Figure 12a, five streaklines reached and passed the line segment between the leftmost and rightmost chemical sensors. While nine streaklines reached the line segment in the experiment for 45° backward jet discharge, seven reached the line segment in the simulation shown in Figure 12b. However, although the left and right adjacent streklines in Figure 12b did not reach the line segment, they passed just slightly outside the leftmost and rightmost sensors. The streaklines that reached the line segment between the leftmost and rightmost sensors show the trajectories of water samples collected to the array of sensors. When jets were discharged perpendicularly to the sides, only five streaklines reached the sensors in both simulation and experiment. Therefore, water samples can be collected only from a narrow frontal region for this jet discharge. When jets were discharged 45° backward, water samples are collected from a wider angular range: seven streaklines in the simulation (nine in the experiment) reached the sensors. These results indicate that the flow fields calculated by the CFD simulations can be used to evaluate the angular range of water sample collection to the chemical sensors, although there still are small discrepancies between the simulations and experiments. In Figure 12, we considered the case of four electrochemical sensors placed at the fixed locations. However, the simulation results can also be used to optimize the number of sensors and to design chemical sensor arrangement around the jet discharge device.

## 4. Conclusions

In this paper, we reported the results of simulations conducted using RANS realizable *k*-*ε* turbulence models. With the improved grid division and enlarged calculation domain size, the flow fields that match well to the empirical velocity profile equations and flow visualization experiments were produced. We conducted simulations in two representative directions of the jets, i.e., perpendicularly to both sides and 45° backward. The angular range of water sample collection to the four chemical sensors placed in front of the jet discharge device was successfully evaluated for these two jet discharge directions. Simulation techniques presented in this paper can be applied for developing jet-assisted chemical sample collection devices and accomplishing efficient chemical sensing in autonomous underwater vehicles. Although each simulation takes approximately a week, the calculations can be sped up using parallel graphics processing units or cloud computing. Such simulation results are useful for designing a jet discharge device and sensor arrangement for specific sensing applications.

In the case of crayfish, the velocity of the inflow generated by the jet discharge is only 5 mm/s at 10 mm from a crayfish [13]. Since it takes some time to draw chemical samples from distant places with this inflow velocity, the device under consideration would find more attraction in flows with smaller scales. However, larger systems can also benefit from the jet discharge. Whenever a suction pump is used to collect chemical samples, you always need to release the sucked water somewhere. The chemical sample collection efficiency can be increased by releasing the sucked water from jet discharge ports. No additional energy is required for this jet discharge. In our flow visualization experiments, water was sucked into the frontal inlet port of the jet discharge device at 300 mL/min. The time to draw an ink blob released at 20 mm from the device to the sensors by this suction alone was 4.2 s. When the sucked water was discharged perpendicularly to the sides as shown in Figure 10a, the time was reduced to 2.4 s. In this case, 43% reduction in water sample collection time was achieved simply by discharging the sucked water to the sides. Similar examples can also be found in the literature on local exhaust ventilation hoods. Jet discharge increases the velocity of inflow to the exhaust hood, and therefore enhances the distance over which dust can be collected. It was reported in [17] that the reach of a low-pressure round suction inlet was increased by a factor of 3 when 40% of the sucked air was ejected from a narrow annular split to form a radial jet around the perimeter of the suction inlet.

The study on jet-induced chemical sample collection is also of biological interest. The distance and angular range of chemical sample collection during the foraging behavior of crayfish has not yet been fully revealed. We believe that this paper will serve as a starting point and will lead to further investigations for obtaining full understandings of crayfish behavior.

Further comparison between the results of the simulations and flow visualization has revealed that there are 20–35% differences in the magnitudes of the flow velocity vectors. By conducting CFD simulations with different boundary conditions, it has later turned out that this discrepancy has been caused by the limited size of the water container that was used in the experiments. The results of this investigation will be reported in a different paper. Crayfish might be adaptively changing the directions of the jets during their olfactory food search, in order to vary the direction of the inflow to themselves. Future work will be addressed to assess the effectiveness of changing the directions of the jets during the foraging walk.

## Figures and Tables

**Table 1:** Simulation setups in the previous work.

	Jet Discharge to the Sides	Jet Discharge to 45° Backward
Computational domain	568 mm in width, 284 mm in depth, and 100 mm in height	284 mm in width, 284 mm in depth, and 100 mm in height
Total number of cells	786,298 (Growth rate = 1.16250)	940,068 (Growth rate = 1.16250)

**Table 2:** Revised simulation setups.

	Jet Discharge to the Sides	Jet Discharge to 45° Backward
Computational domain	2100 mm in width, 2100 mm in depth, and 100 mm in height	2100 mm in width, 2100 mm in depth, and 100 mm in height
Total number of cells	11,247,418 (Growth rate = 1.06)	8,022,878 (Growth rate = 1.06)
